# Hamster numbers: biopolitics and animal agency in the Dutch fields, circa 1870-present

**DOI:** 10.1007/s40656-021-00398-3

**Published:** 2021-03-30

**Authors:** Raf De Bont

**Affiliations:** History Department, Faculty of Arts and Social Sciences, Chair of the History of Science and the Environment, Maastricht University, Grote Gracht 80-82, 6200 MD Maastricht, The Netherlands

## Abstract

Numbers of European hamsters (*Cricetus cricetus*) in the Dutch Province of Limburg have been subject to much scrutiny and controversy. In the late nineteenth century, policymakers who considered them too numerous (and invasive) set up eradication programs. In the second half of the twentieth century, even when its domestic relative (*Mesocricetus auratus*) increasingly circulated as a pet in urban spaces, the numbers of European hamsters in the rural areas collapsed. Large-scale preservation campaigns and reintroduction programs ensued. According to some media, all this has turned the European hamster into the most expensive undomesticated animal of the Netherlands. A whole network of institutions became involved to save the species – ranging from local activist organizations, over zoos and universities, to federal ministries and international organizations. The interactions between the Dutch and ‘their’ hamsters, this article argues, were inscribed in various forms of biopolitics. The article highlights the changing discursive framings and spatial practices that have shaped the management of *Cricetus cricetus* over time and calls attention to the diversity of living and non-living agents that produced the multispecies choreographies of the present-day Limburg landscape. Finally, it alerts us to the (sometimes-paradoxical) kinds of agency that reside in the numbers of non-human animals.

## Introduction

The European hamster (*Cricetus cricetus*) seems an improbable object of controversy. Unlike its better-known relative the golden hamster *(Mesocricetus auratus)*, which has become a popular house pet, the European hamster largely carries out its existence far away from human sight. A nocturnal or crepuscular animal, it spends most of its day in its underground burrow in the fields. Between October and March, furthermore, the species’ hibernation renders it completely invisible for even the most devoted of researchers. Indeed, people can live close to sizable populations of European hamsters without ever seeing one single individual. Yet, in the Netherlands – a country at the western edge of the hamster’s current range – the secretive species has raised emotional discussions at several occasions. These discussions concerned the question how to control the hamster numbers and its geographical spread. In the late nineteenth century, when farmers first spotted European hamsters in the southern province of Limburg, the Dutch deemed this question particularly important in the context of pest management. From the 1960s onward, the interest in managing the species’ numbers and expanse returned to the agenda, but this time in the context of regional nature protection policies. In this period, projects of extermination gave way to (heavily discussed) projects of conservation. While the European hamster remained physically mostly invisible, the species came to be ubiquitous in the Dutch public debate.

Invisible yet ubiquitous, the European hamster of Limburg offers a good starting point to discuss changing human-animal interactions in the Anthropocene. In human-dominated landscapes like that of nineteenth- and twentieth-century Limburg, non-human animals have become subject to various managerial regimes. This article will therefore explore the interactions between the Dutch and ‘their’ hamsters as a form of biopolitics in the making. While Foucault coined the term biopolitics to understand the ways in which *humans* are governed at the population level (Foucault 2008; Lemke 2011, pp. 33–52), various scholars have extended the concept to include the management of non-human animals (Biermann et al. 2017; Hodgetts 2017; Lorimer 2015, pp. 57–76). This recent scholarship has notably highlighted the multiplicity of the logics and techniques through which animal bodies are regulated. The question indeed of ‘which lives must be fostered and who or what is killable and why’ has typically been the topic of lively debates (Biermann et al. 2017, p. 2). In the Netherlands, this was certainly the case for the European hamster.

This article addresses the discursive framings and spatial practices that shaped the management of ‘wild’ hamsters in the Netherlands since the late nineteenth century. As such, it conceives biopolitics broadly as involving both conceptual and practical work. Biopolitics encompasses valuing hamsters (their role, position and behavior), imagining spaces (where these hamsters belong or do not belong) and conceptualizing populations (their relevant criteria and optimal numbers). It also involves concrete techniques of monitoring, breeding, circulating, safeguarding, policing and killing. These conceptual and practical doings – which co-construct each other – mobilized a wide variety of agents. They include the administrators of the nation state and state-sanctioned experts that have originally been highlighted in studies of modern biopolitics. Yet, this article will argue they also involve journalists, activists, lawyers and farmers, as well as officials of regional and supranational institutions.

While scholars have used the concept of biopolitics to lay bare the exercise of power over particular subjects, the notion does not entail that the latter become ‘fully determined by technologies of control’(Bröckling et al. 2011, p. 14). The subjects of biopolitics are never without ‘agency’ – even when they are hamsters. Over the past two decades, scholars in animal history and human-animal studies have been particularly occupied with theorizing the notion of animal agency. Originally, this resulted in widely divergent understandings of the concept. Yet, under influence of actor-network theorists such as Bruno Latour and Michel Callon (Callon 1986; Latour 2007), a growing consensus emerges around broad definitions in which animal agency is understood as a capacity to ‘make a difference’ – either as an individual or as a species (Fudge 2017; Pearson 2017; Shaw 2013; Weil 2012). Such agency, then, has, inter alia, been sought in the ways through which animals ‘destabilize, transgress, or even resist our human orderings’ (Philo et al. 2000, p. 5). Some scholars have added that beyond an unwillingness to ‘submit to human authority’, the agency of animals can equally be found in the ways they ‘influence, enable and sustain human intentions and activities’ (Pearson 2015, p. 713; Pooley-Ebert 2015; Rees 2017, p. 128). Integrating these insights into the study of biopolitics implies an attention for ‘the material realities of all live’ (Asdal et al. 2017, p. 25). For the case under consideration, this means that alongside the contributions of various human actors, we pay heed to the ways hamsters escape, resist or enable biopolitical governance. Given that this governance – either aiming for the animals’ extermination or preservation – mostly focused on controlling numbers (Cushing et al. 2018), this article will argue that it is in their quantity that an important part of the hamsters’ agency can be sought. Populating particular areas or disappearing from them, hamsters voted with their feet.

Most studies on the biopolitics of wildlife management concern contemporary discussions. The European hamster in the Netherlands, however, offers a case that concerns long-term developments in human-animal interaction over a period that stretched back a century and a half. During this whole period, the hamster’s space of belonging was an object of discussion. Originally an inhabitant of the Eurasian steppes, *Cricetus cricetus* took advantage of the Neolithic revolution following the expansion of agriculture to the west (Niethammer 1982). According to some, it only reached the Netherlands in the late nineteenth century. As a ‘culture follower’, whose arrival is of unclear dating, the European hamster in the Netherlands transcended straightforward dichotomies between ‘alien’ and ‘native’, ‘wild’ and ‘domesticated’, ‘nature’ and ‘culture’. This liminal position certainly allowed for a multiplicity of potential biopolitical discourses and practices. These discourses and practices, furthermore, radically changed over time.

## A Monstrous Alien in Agricultural Land

The appearance of the European hamster in the Dutch written record is rather sudden. Only in the late 1870s was the species noticed by (subsequently) journalists, policymakers and zoologists. In November 1877, the national newspaper *De Tijd* ran a short piece indicating that, while zoologists classified them as ‘alien’(‘*uitheemsch*’), farmers had spotted hamsters in the village of Wylre in south-eastern Limburg (“No Title,” 1877a). A few days later, another newspaper brought a story from the same village, where a dog had dug up and ‘slaughtered’ one of these alien animals. The same article stressed that the species was already causing quite some damage to the grain harvests of the local farmers (“No Title,” 1877b). Until the summer of 1879, there was no further hamster coverage, but then suddenly the animal seemed to be everywhere. Several newspapers brought stories on ‘the most notorious grain thieves of the rodent family’. The articles wrote about ‘colonists’, resembling ‘big rats’, which were seen all across Southern Limburg and rapidly increasing in numbers (“No Title,” 1879a; “No Title,” 1879b). The Amsterdam-based *Algemeen Handelsblad* dug deeper into the subject and reported that the earliest Dutch sightings of hamsters had been around 1870 in a village near the Dutch-German border. In line with this finding, the journal claimed that ‘one generally considers that the grain destroyers have come to us via Germany’(“No Title,” 1879c, p. 1). By this point, the media narrative had been set: the hamster was an alien threat to Dutch agriculture.

Scientists and policymakers ‘discovered’ the hamster only in the wake of the press. The first Dutch zoologist to mention its presence on national territory was the Rotterdam zoo director Adriaan van Bemmelen, who, in November 1879, addressed the issue at the yearly meeting of the Dutch Zoological Society in The Hague. Yet, his intervention gave little evidence of direct observation and mostly limited itself to summarizing the recent discussions in the media (“Verslag van de gewone huishoudelijke vergadering,” 1881). In the meantime, also policymakers in the capital were up to speed – once again, probably, through the coverage in the dailies. During a national budget control, the appearance of the ‘highly dangerous grain-eating animal’ was raised and, as a result, the Minister of Public Works, Trade and Industry requested further data from the Governor of Limburg (Husson 1949, p. 26). A systematic consultation of the latter with the Province’s mayors resulted in hamster notifications from at least twenty-eight municipalities. The Royal Commissioner in the Province responded with a missive in December 1879. The letter stressed that the invading rodent had not caused all too serious damage yet, but that given its reproduction rate it could soon prove ‘a disaster for agriculture’. The Commissioner therefore called upon the local authorities to take urgent measures for its ‘extermination’(“De Hamster” 1880, p. 1; Husson 1949, pp. 26–29). The logic of his biopolitical program was clear.

Most 1870s sources implied the hamster only recently incurred from Germany, but some scientists have later cast doubt on this claim, arguing its presence in the area should probably be dated back several thousands of years. (Clason 2002; Weber 1919). They invoke the hamsters’ evasive habits and confusion over rodents’ taxonomy among the population to explain their centuries-long invisibility. This explanation, however, does not seem to account for the westward expansion noticed by contemporaries in the 1870s and the following decades (“De Hamster” 1880; “No Title” 1879c). After all, through the 1880s and 1890s, hamster sightings moved further west across the Belgian border to eventually reach the outskirts of Brussels in the 1930s and 1940s (Dupond 1932; Leplae 1899; Libois et al. 1982). In any case, while in the second half of the twentieth century most zoologists would leave the question whether the hamster was a Dutch ‘indigenous’ species open (Husson 1949; Thissen 2002), commentators in the late-nineteenth century showed less qualms. They unambiguously qualified it as ‘alien’.

While the label of ‘alien species’ certainly helped in the construction of the hamster as a threat, actual hamster numbers and the perceived damage these caused were, of course, also critically significant. To understand how both the actual numbers and their perception changed, developments in local and global agricultural practice are of crucial importance. In the late nineteenth century, Limburg farmers had left the three-field system behind, creating fields on which grain, in combination with a variety of other crops, could be harvested every year. Yields, furthermore, increased through seed improvement and new fertilization methods, while field size remained small and mechanization limited. As such, hamsters both had year-round cover and food. All this can certainly help to explain the sudden explosion of hamster populations in the 1880s (Pelzers et al. 1984). While Limburg farmers unconsciously constructed the perfect hamster habitat, they were also increasingly sensitive to the potential damage these rodents might cause. Limburg, after all, was strongly impacted by the European-wide agricultural crisis triggered by cheap grain imports from the US and Canada (Philips et al. 1965, pp. 209–215). In such a context, farmers were receptive to protect their harvest from rodents who according to some media collected up to hundred kilogram per family in their burrows (“No Title,” 1879c). Importantly, in the same period Dutch policymakers gradually moved away from traditional *laissez faire* ideals and proved willing to intervene more actively in national agricultural production (Koning 1994, p. 94). Hamster control was one field of potential policy intervention.

At the explicit request of the Royal Commissioner, municipalities introduced bounties for killed hamsters from 1880 onward (“De Hamster” 1880; “No Title” 1881). Not all municipalities of southern Limburg were involved, however and the numbers of hamsters brought in fluctuated strongly depending on time and place. Even in years of local population explosions, the figures remained limited to a few hundred per municipality – far less than the several thousands of hamsters killed in some villages in neighbouring Belgium and Germany (Husson 1949, pp. 29–37; Pelzers et al. 1984, pp. 207–208). While this suggests the gravity of the problem might have been comparatively limited, both journalists and agricultural experts perpetuated an image of imminent menace, constantly incentivizing farmers to exterminate the species on their land. Newspapers and expert publications gave advice on possible methods of destruction, including smoking hamsters out of their burrows with burning sulphur-dusted rags, drowning them, poisoning them, trapping them, catching them with ferrets, or digging them up with the help of rat dogs (Leplae 1899, pp. 476–479; “No Title” 1880; Ritzema Bos 1891, p. 82; Staes 1899, pp. 190–192). According to the Belgian agricultural scientist Edmond Leplae, whose work also appeared in the Netherlands, the last method was to be preferred. In his view, after all, it contained ‘a certain sportive aspect’(Leplae 1899, p. 478).

The fact that, in the late nineteenth and early twentieth century, the control of ‘pest’ animals grew into an object of special expertise fitted in with broader transnational developments. Thanks to an increased interest of state authorities, several western countries witnessed the rise of applied agricultural sciences (Clark 2001; Jansen 2003; Sayer 2017). In the Netherlands, the biologist Jan Ritzema Bos played a crucial role in the discipline-building. Initially a teacher at the State agricultural school, Ritzema Bos would become the first director of the Psychopathology Laboratory at the University of Amsterdam in 1895 and, from 1899 onward, at the national Psychopathological Service (Maat 2001, p. 52). In all these capacities, he tried to institutionalize the study of agricultural pests – with, among them, hamsters.

The European hamster was on Ritzema Bos’s radar as early as 1880, when he interfered in the press to clear up what he saw as taxonomic confusion among local farmers. He indicated the latter commonly mixed up hamsters with water voles – a confusion he believed interfered with an efficient control of both pest species (Ritzema Bos 1880). For the identification of water voles Ritzema Bos could refer to his own newly published textbook *Landbouwdierkunde [Agricultural zoology].* The hamster, however, was not included yet (Ritzema Bos 1879). During the following decades, he gradually made up for the oversight. In 1891, he discussed the species as a ‘harmful rodent’ in his influential *Tierische Schädlinge und Nützlinge [Harmful and Useful Animals],* also including advice on its extermination (Ritzema Bos 1891, pp. 80–82)*.* From then onward, the categorization of the hamster as a pest echoed in Dutch handbooks for secondary schools, gymnasia, agricultural state schools and normal schools (Boerman et al. 1920, p. 123; Hoogeveen et al. 1919, p. 66; Horn et al. 1924, p. 207; Ritzema Bos 1902, pp. 63–65; Van der Wijk 1931, p. 105). Ritzema Bos, furthermore, prominently featured the hamster in a series of school wall charts devoted to ‘animals harmful for agriculture’ (*Nederlandsche *Schoolplaten 1922, p. 52). In just a few decades time, the European hamster had become fully incorporated in a wider Dutch educational regime that had to alert the nation to the dangers of unruly animals. In this regime, the countryside was defined as a space of efficient agricultural production, in which species such as the hamster – both ‘harmful’ and ‘alien’ – had no place.

The transnational development of state-sanctioned agricultural science went hand in hand with a pestilence discourse that was internationally quite homogeneous. Such a discourse – whether it concerned prairie dogs in the US or grape *phylloxera* in Germany – typically framed ‘harmful’ species as aggressive, breeding out of control and encroaching upon human space (Jansen 2003; Jones 1999; Knight 2000). Late-nineteenth and early- to mid-twentieth century discussions of the European hamster in both the Netherlands and bordering Belgium aligned with this international trend. Like in the pestilence discourse that developed elsewhere, military metaphor was often prominent (Russell 1996). The European hamster was deemed an ‘enemy’, its movements described as ‘invasions’ or ‘attacks’ and its living spaces as ‘occupied territory’. Maps, based on information provided by local mole-catchers, farmers and counselors, showed their advances like those of enemy militias (Leplae 1899; Staes 1899). Leplae explicitly described the purportedly progressing hamsters as an ‘army of rats’ (Leplae 1899, p. 475). In this way, he not only tapped into images of military threat, but also linked the hamster to another rodent species that was a longstanding bearer of negative associations with filth, infection and economic peril (Burt 2006).

The discourse of threat not only concerned the numbers of the European hamster, but also its behavioral traits. In the decades around 1900, journalistic descriptions dwelled on the species’ perceived aggressiveness, typically illustrated by anecdotes of attacks on dogs, horses and humans (Dixi 1919). An early-twentieth-century article in the *Nieuwe Tilburgse Courant* described assaults by hamsters ‘as big as a cat’ and considered the species ‘extremely dangerous’(“Brieven uit Brussel” 1902). In the 1930s, then, several dailies covered a story of a priest, who was bitten in the knee by a hamster while on an evening walk in Southern Limburg. According to one newspaper, ‘the animal did not turn a hair until it was kicked to death’ (“De hamster nuttig of schadelijk” 1937, p. 3). While often just casually alluded to, such aggression occasionally also led to further reflections on the animal’s psychology. One such reflection, published in the *Bataviaasch Nieuwsblad* in 1911, deserves full quotation here, as it nicely illustrates how the pestilence discourse mixed psychologizing tendencies with xenophobia. It reads:

‘The longer the friend of nature studies this monster in the shape of a rodent, the more he is convinced that underneath this colorful gambeson is beating a dark heart, in which eccentric egoism, shameful malice and wrathful hate against all its fellow creatures seems to have suffocated every tender feeling. […] It seems as if nature has marked this criminal. In its outward appearance, it has nothing of the gracefulness and the cheerfulness of past native rodents and its broad face with its piercing eyes, the strongly developed lower parts and the always threateningly exposed violent teeth unmistakably contribute to an expression of nastiness and hatred’ (“Een klein monster” 1911, p. 13).

Hamsters, thus, were like foreign criminals, both alien and aggressive.

The allegory, furthermore, worked in two directions. Dutch journalists not only likened hamsters to particular types of humans, but they also described the behavior of particular humans by reference to hamsters. While the British turned ‘badger’ into a verb in order to refer to pestering (Cassidy 2019, 25), the Dutch started to use ‘hamstering’ (‘*hamsteren’*) as a synonym for hoarding. The term shows up for the first time in newspapers during the First World War, to rise in popularity during the Second World War and again during the heating up of the Cold War after 1948 (“De Hamster” 1918; “Hamsteren” 1939; “Het hamsteren der hamster” 1939; Dixi 1919; F.S 1950). During such periods of crisis and scarcity, ‘hamstering’ was used specifically to denote the secret and illegal stashing of large stocks of foodstuffs. In those contexts, references to the much-maligned hamster were supposed to have a moralizing effect. After all, as *De Heerenveensche Koerier* had it in 1950, ‘when honestly looking into the mirror’, we do not want to see ‘the traits of the rodent with the cheek pouches’ (F.S 1950, p. 1). Thus, the rhetoric of hamster control in landscapes of agricultural production reverberated in the moral economy of human food provision.

The metaphorical use of ‘hamstering’ clearly echoed the biopolitics of extermination that had taken shape in the Limburg fields from the 1870s onward. The practices and rhetoric of this biopolitical regime had been co-shaped by a wide range of actors including farmers, politicians, agricultural scientists and journalists. The story, often cast in military terms, would not be complete, however, without acknowledging the agency of their opponents in ‘warfare’: the hamsters. It had, of course, been their population explosions that made them into a matter of biopolitical concern in the first place. And despite the self-confident language of agricultural science, bringing about hamster extermination did not prove easy. To perpetuate the military metaphor, farmers and scientists seemed faced with guerilla rather than conventional warfare. Reading the sources against the grain, it becomes clear that the hamsters’ underground and secretive habits made it very difficult to turn them into objects of knowledge and control (Husson 1949, p. 50; Ritzema Bos 1891, p. 81). They continued their westward expansion for decades, leaving scholars puzzled as to how they had managed to overcome major geographical barriers such as the river Meuse (Dupond 1932; Leplae 1899). Pest measures clearly did not prevent the colonization of new territory. To be true, in the twentieth century hamster numbers went down, but later analyses indicated this should be explained by changing agricultural land use rather than extermination campaigns (Libois et al. 1982). In any case, by *not* going extinct, the Limburg hamsters showed the limits of the biopolitical interventions that were designed for exactly that purpose.

## *Intermezzo:* A Companion Species in the Tower Blocks

By the time that *Cricetus cricetus* had become the symbol of Cold War stashing, it was no longer the only alien hamster species on Dutch soil. The Post-World War II period, after all, also saw the advent of its smaller relative: the Syrian or golden hamster. This species did not enter the country as an agricultural pest, but as a much beloved pet. Given that its reception could hardly have been more different than that of its wild Limburg counterpart, the ‘invasion’ of the golden hamster deserves some attention here.

The Syrian hamster resembled its distant relative in that it also originated from steppe habitats. Yet, its journey to Western Europe was of a different kind altogether. It started in the 1920s, when Saul Adler, a parasitologist at the Hebrew University of Jerusalem, was looking for a lab animal for the study of *leishmaniasis.* The species typically used for such research was the grey dwarf hamster (*Cricetulus migratorius*), but, since this needed to be imported from the Far East, Adler sought a local substitute. Therefore, he sent out his zoologist-colleague Israel Aharoni on an expedition to the Syrian steppe-lands, where, with the help of local guides, the latter managed to find and catch one female Syrian hamster with thirteen young. In the process of catching, transporting and caging, most individuals either died or escaped, so that (depending on the source) only three or four individuals remained. These, however, bred particularly well in captivity and, in the words of Aharoni, proved ‘convenient for endless laboratory experimentation’(Murphy 1985, p. 12). Over the following years, Adler eagerly spread his model organism. In 1932, he introduced Syrian hamsters to Britain (purportedly by smuggling them in the country in his coat pockets) and, from 1938 onward, to the United States as well. In this way, his handful of individuals gave rise to a rapidly growing and globe-spanning population of lab animals (Adler 1948; Murphy 1985; Yerganian 1972). And the laboratory was not the only man-made space in which they thrived. In the 1940s, hamster fanciers in both Britain and the US successfully turned the quick-breeding and docile rodent into a popular indoor pet (Grier 2006, p. 41). In just over a decade, ‘domesticated’ golden hamsters easily outnumbered the small ‘wild’ populations in (then still) the French Mandate for Syria and Lebanon.

In the Netherlands, the presence of golden hamsters can be traced back to at least 1946, when a trader started importing them after a visit to a London pet exhibition (“Syrische hamsters” 1957). It took another decade until they became truly popular, but by 1957 the craze for the newest ‘American fashion animal’ caught on (“De goudhamster” 1956, p.13). Interestingly, journalists linked its popularity to post-war urbanization and public housing development. Living in small terraria, Syrian hamsters were presented as well-suited to a time of ‘modern’ tower blocks, where the keeping of larger pets was often impossible or forbidden (“De goudhamster” 1956; Goudhamsters” 1957). Since they slept during the day, hardly made any sounds and did not smell, they were considered ‘the ideal room-mates for bachelors with strict land ladies’(“Hospita vermoedt niets” 1956, p. 3). Journalists complimented Syrian hamsters for their ‘pleasant manners and funny appearance’(“Hospita vermoedt niets” 1956, p. 3) and described them as ‘sweet and caring little animals, well-behaved and with a touching defenselessness’(“Leeuwarden kent ze nu ook” 1956, p. 7). Occasionally, journalists further developed this image by comparing the Syrian hamster to their distant relatives in Limburg. A journalist of the Social-Democratic newspaper *Het Volk,* for instance, warned his readers certainly not to mistake the latter for pets, given their aggressive biting habits and great skills at breaking out. ‘It is not recommended’, he or she continued, ‘to start breeding harmful animals that will escape at the first opportunity’(“Hospita vermoedt niets,” 1956, p. 3).

The Syrian hamster of the newly built tower blocks, thus, became the antipode of the European hamster that inhabited the equally modern space of the Limburg grain fields. It was not unruly and threatening, but well-controlled and domestic. Contained within the space of urban home life, it did not interfere with agricultural production. It remained out of the remit of the biopolitics of state-sanctioned agricultural science, circulating in a circuit of private fanciers. Its prolific breeding as well as its placid character and limited space requirements were assets in this context and proved crucial for their commodification. The Syrian hamsters’ physical and behavioral attributes thus tied in with the logic of the pet industry. In various ways these attributes indeed *enabled* the industry.

As a commodity and cosmopolitan companion species the Syrian hamster freely crossed borders. Unlike the arrival of the European hamster, its entering of the Dutch national territory was therefore not likened to a military invasion. The Syrian hamster, newspapers assured their readers, came with ‘peaceful intentions’(“Leeuwarden kent ze nu ook” 1956, p. 7).

## A Local Rarity in a Disappearing Habitat

While hamster pets were conquering Dutch housing developments, the wild hamsters in the fields of Limburg were in decline. This plainly came to light in a survey of 1960 held by the National Institute of Field Biological Research for Nature Preservation [*Rijksinstituut voor Veldbiologisch Onderzoek ten behoeve van Natuurbehoud*, RIVON]. As part of a larger national scheme to map the geographical spread of mammals, the Institute sent out two young biologists to chart hamster burrows. One of the biologists in question gained renown for his ability to locate these burrows, riding a moped through the fields and tracing the hamsters with his self-trained Dachshund (W. van Mourik, personal communication, March 12, 2020). His inventory showed that, while the overall hamster territory remained largely unchanged, their numbers clearly went down rapidly. The eventual RIVON report speculated that changes in agricultural practice and predation by dogs and cats were to blame for this trend. It also indicated that the time might have come to start thinking about the creation of hamster reserves (Glas 1961; Mourik et al. 1962).

The decline of hamster populations in the 1960s and 1970s was accompanied by a shift in representation. Following the observations of RIVON researchers, some newspaper articles indicated that the hamster winter stocks did not contain hundred kilos of grain as often had been proclaimed, but only a mere five kilograms. Furthermore, in line with this reassessment, the hamster was gradually rebranded from a ‘harmful’ into a ‘useful’ animal, pointing particularly to its role in destructing ‘real’ pests such as insects and mice. Finally, journalists abandoned the old frame of the species’ German provenance and, thus, the hamster’s invasive status. Increasingly, the European hamster appeared in the national and regional media as a rare indigenous animal, typical of southern Limburg, that contributed to the appealing natural distinctiveness of the peripheral province (Haimon 1974; “Hamsters” 1963; “Hamsterstand” 1964; “Zuid-Limburg” 1969).

Such representational changes occurred against a background of rising environmental consciousness in the Netherlands. The period around 1970 witnessed the foundation of new Dutch conservation organizations and institutes, while existing ones (such as RIVON) gained more influence and leverage (Van Der Windt et al. 2009). Televised nature documentaries, furthermore, contributed to the shift in mentality and a revaluation of Dutch wildlife. Between 1968 and 1998, two Limburg film-makers shot no less than 240 episodes of a series entitled ‘Nature at home’ [*Natuur in eigen Land*] to be broadcasted on national television (Hogenkamp et al. 2009). Their footage included ‘most remarkable’ shots of hamsters ‘still to be found in the wild’(“Limburgse Natuurzondagmiddag” 1980).

While the representation of hamsters thus started to change from the 1960s onward, it took until the 1990s before action on the ground materialized. A new survey, carried out in 1994 by the Limburg Natural History Society in collaboration with a consultancy firm, showed a complete implosion of the population. Hamsters proved unable to keep up their numbers in a landscape in which farmers cultivated less grain (and more maize) and used increasingly efficient harvesting techniques (leaving less food) (Krekels et al. 1996). With the resulting implosion of hamster populations we see a shift in agency. Their numbers became less relevant as a threat for agricultural production, but more for the emotion of loss they instilled among some parts of Dutch society. Imploding numbers could indeed ‘make a difference’ as much as exploding ones and it did not take long for the hamster to again mobilize a great variety people.

In 1996, the national government selected the hamster as one of thirty key species in its so-called Action Plan for Species Policy [*Plan van aanpak soortenbeleid*] (Krekels 1999, p. 5). Despite this early governmental interest, however, most commentators indicate that it was particularly the highly visible activism of a newly founded society that got things moving. The society in question was called Badger and Tree [*Das en Boom*] and had been established in 1981 to stem the decline of Dutch badger populations. In the late 1990s, the Society’s mediagenic president Jaap Dirkmaat decided to partially refocus his organization’s attention to hamsters after a registrar suggested him that their stricter protection status would offer more legal leverage. The European hamster’s listing on both the Council of Europe’s Bern Convention and the EU Habitat Directive indeed enabled Dirkmaat to institute summary proceedings against the Dutch government for not fulfilling its international commitments. This was only possible because, in the latter decades of the twentieth century, the European institutions had emerged as major players in environmental policymaking (Boardman 2006). Yet, while international institutions such as the EU had the power to collect substantial fines in case member states broke their agreements (and particularly when protected animals went extinct on their territory), it depended on private actors to bring the infringements before the court. This was exactly what Dirkmaat decided to do (J. Dirkmaat, personal communication, March 9, 2020).

*Das en Boom* started its actions with legal protests against a planned highway, the A73, which would run through hamster habitat. The media-savvy Dirkmaat explained in the press that the Dutch government all too easily criticized the French for shooting their Pyrenean brown bears, while neglecting their duties towards animals with equal protection status on their own territory (Postma 1996). A whole range of court cases ensued. The most visible of these would be litigations brought before the Council of State against an EU-subsidized business park at the Dutch-German border. Employers’ organizations argued there were actually no hamsters in the area of the projected park, complaining that economic development was halted for nothing but ‘ghost animals’ (“Limburgse werkgever” 2000). As the Council of State annulled the authorisation for the park twice, it looked like EU environmental legislation was about to torpedo plans of the same EU for cross-boundary industrial development. Yet, eventually, the court ruled in favour of the business park (Bastmeijer et al. 2003, pp. 57–60; “Leefgebied” 2000; Slepcevic 2009, pp. 204–206). By then, the media could argue ‘the poor hamster’ had been ‘more often in the news than that it was sighted in nature’(Ledegang 2001). As early as 1999, *Das en Boom* indicated the species was virtually extinct on Dutch territory. Dirkmaat, then, organized a wake on the field of what were purportedly the last three hamster burrows in the Netherlands. A few semi-celebrities alternated keeping watch in a caravan and three Dutch flags were lowered to half-staff. All this made the national evening news (Greven 1999).

With the old biopolitics of extermination petering out, the period between the 1960s and 1999 saw a rethinking of the place of the European hamster in the Limburg landscape. From an alien pest in a land of production, activists and zoologists turned the hamster into a marker of a disappearing traditional countryside. Its presence was still monitored through maps, but these no longer indicated an approaching enemy. Rather they represented vanishing populations that needed protection. In an attempt to stop the decline, activists and zoologists tried to renegotiate the hamster’s position along three different geographical scales. At the scale of the province, they mobilized the European hamster for regional identity-building. At the interacting scales of the nation-state and the supranational EU, then, they exploited legal commitments that concerned the continued presence of the species on the national territory. Eventually, the latter commitment would also legitimize its removal from its natural habitat and the setting up of a captive breeding program. A new form of hamster biopolitics was about to start.

## Genes in a Breeding Box

In 1999, in an ultimate bid to save the species, *Das en Boom* requested permission from the Secretary of State of Agriculture, Nature and Food Quality to catch the last individuals in order to start a scheme of ex situ breeding. This was controversial. The local press played into the regionalist feelings of the inhabitants of Limburg (who see themselves as quite distinct from the rest of the country) and represented the plans of *Das en Boom* to move the last remaining hamsters outside of the province as a form of ‘neo-colonialism’ (Dohmen 1999). More importantly, however, like in the case of other captive breeding projects (Alagona 2004), the initiative also proved highly divisive in the nature protection community itself. Some preservationist and animal rights societies (notably *Faunabescherming* and *Dierenbescherming*) believed the capture would traumatize the animals, lead to infertility and, thus, hasten extinction (Dohmen 1999). The societies in question went to court to stop the seizure of the animals, but lost their case. In the spring of 1999, Dirkmaat could thus organize the capture of the ‘last three’ hamsters, which he subsequently introduced to journalists as ‘Adam’, ‘Eve’ and ‘Maria’ (Fokken 1999). In this way, he used an established preservationist strategy of giving animal subjects human names in order to provide them with distinctive characters for the purpose of storytelling in the mass media (Benson 2016, p. 114). Notably the biblical names of Adam and Eve were meant to instil hopes that the hamster couple would be equally successful in replenishing the earth. The hopes, however, were smashed when ‘Adam’ turned out to have cancer and died (Maas 1999). In a bit of a volte-face, Dirkmaat admitted the three hamsters in his possession had not been the last ones after all and *Das en Boom* received permission to catch any other remaining individual that might be left in the wild. Twelve more hamsters were traced and brought in for the breeding program (Lammerse 1999).

Mostly an activist organization, *Das en Boom* did not have in-house expertise regarding the breeding of hamsters. In order to ‘practice’, they performed a trial with European hamsters imported from the Czech Republic, but this turned out to be unsuccessful. An involved zoologist of the University of Wageningen indicated they only found out afterwards that 13 out of the 14 Czech individuals in the trial were male (G. Müskens, personal communication, February 6, 2020). Yet, notwithstanding the unsuccessful trial, the breeding program with captured Limburg individuals did go ahead at *Das en Boom*’s headquarters in Beek-Ubbergen. Once started, the learning curve was steep. After some incidents with aggressive females, a system was introduced in which male hamsters could approach females through a ‘guillotine door’. Furthermore, keepers always had water sprays at hand to ward off females, so that the males could escape after mating (J. Dirkmaat, personal communication, March 9, 2020).

Relatively soon, the breeding procedures were streamlined and the program institutionalized. The government set up a breeding task force, which apart from *Das en Boom*, consisted of Wageningen biologists, the leading Dutch preservation organization *Natuurmonumenten and* the Rotterdam Zoo (Krekels 1999, 26). In order to spread the risk in case of fire or epidemic, the zoo agreed to house half of the individuals (Lammerse 1999). Despite some initial setbacks and improvising, this multi-stakeholder arrangement proved to be a success. The hamsters were held in ‘breeding boxes’, in which, with the exception of short and highly controlled mating sessions, they lived alone. Moved to this new spatial context – not unlike the terrarium of the ‘domesticated’ Syrian hamster – the breeding picked up. The unexperienced breeders gradually learned that the females went into heat every fourth day (“Seks korenwolf nog steeds niet succesvol” 2000). In the wild they would typically have two or three litters a year, but in captivity this number could eventually be increased to five (“Gefokte korenwolven gaan naar Heer” 2000). In the fall of 2001, the captive hamster population had already grown to 135 individuals (“Korenwolven” 2001).

With all remaining Dutch hamsters in a captive breeding program, their procreation became registered in studbooks, turning their genes into an object of calculated human management. The choices to be made in orchestrating hamster genetics were not self-evident, however, and the participants in the task force disagreed over the approach to take. In an initial phase, Dirkmaat showed himself open to the idea to include Slovakian and Czech hamsters (which were still numerous), but Wageningen biologists pushed back, indicating such individuals might evolutionarily be unadapted to Dutch habitats (J. Dirkmaat, personal communication, March 9, 2020). The press picked up on the disagreement. While some voices deemed Dirkmaat’s position a form of ‘fauna-cheating’, others accused the biologists of ‘econazism’ (“Overheid zit korenwolf dwars” 1999). The Wageningen researchers defended their stance with the then generally accepted claim that western hamsters constituted a subspecies that differed from the more numerous European hamsters in Eastern Europe (Mitchell-Jones et al. 1999). From a conservation perspective, such a categorization clearly also offered strategical benefits. Since subspecies are the object of specific international conservation regulations, the status provided preservationists with – as one zoologist formulated it – ‘a legal big stick’ (M. la Haye, personal communication, March 3, 2020). It was, thus, a setback when, in 2004, new genetic research suggested that the hamsters of Western Europe did not make up a separate subspecies after all (Neumann et al. 2004). Yet, while this arguably decreased its international conservation value, the revised status changed little in the approach of the breeding project itself. The hamsters in the region of the Netherlands and bordering Belgium and North Rhine-Westfalia were still considered a separate population, referred to as the ‘BNN population’, which was genetically distinct enough to be kept apart from their less threatened eastern counterparts. As such, the focus continued to be on ‘pure’ and ‘local’ animals (Haye et al. 2005; Haye et al. 2012; Kuiters et al. 2010).

Apart from ideals relating to population genetic *purity*, Dutch hamster breeding was also driven by genetic ideals of *diversity*. Most participants in the taskforce shared concerns over inbreeding – an element that became central to the discursive framing of the European hamster in its western range. Not only was the BNN population considered to be genetically poor to begin with, but to work from a founder population of just 14 closely related animals was believed to be particularly worrisome. Researchers feared such inbreeding could lead to pathology and decreased fertility (M. la Haye, personal communication, March 3, 2020). For that reason, the taskforce sought to increase the genetic variation from their Dutch captive hamsters by including individuals from the rapidly declining populations in Belgium and North Rhine-Westphalia. Yet, what they described as a ‘natural’ unity in terms of population genetics proved to be divided by very real borders of regional and national administrations. Some individuals from Belgium and one from Germany could be added to the Dutch breeding project in 2003 and 2004 (Haye et al. 2005; Haye Koelewijn et al. 2014; Haye Swinnen et al. 2014), but negotiations to come to a fully integrated program led to substantive delay and ultimately got stuck in administrative hassle with the German authorities (B. van Noorden, personal communication, March 12, 2020). In any case, the resulting genetic mixture of genes in the Dutch program was considered diverse enough to be viable. At the same time, geneticists deemed it local and pure enough to preserve its original evolutionary adaptations.

The crossing-in of ‘foreign’ hamsters was the object of much reflection. Geneticists legitimized the practice by claiming it was an equivalent to ‘natural migration’. They, furthermore, closely monitored its effects on ‘genetic recovery’ and ‘fitness’, measuring both on the basis of average litter size. Notably, the genetic material of the single German male added to the breeding program in 2003 proved impactful, starting a breeding line that with an average litter size of 7.2 was significantly higher than the 5.3 of the ‘purely Dutch’ line. While biopolitics moved to the genetic level, the overall goal remained what it had been before: controlling population numbers. The individual referred to in publications as ‘the German (superior) male’ proved highly instrumental in this regard (Haye Koelewijn et al. 2014; Haye Swinnen et al. 2014).

In sum, the 1990s had led to a radical shift in human-hamster relations in the Netherlands. The European hamster was moved from its dwindling ‘natural’ habitat to the human-controlled settings of breeding centres more than hundred kilometres away. The move brought the hamster under the logic of zoo breeding, as such ‘relocating the value of an animal from the space in which the animal lives to the genealogical relations from which it came’ (Friese 2013, p. 123). In the process, monitoring instruments shifted from maps to studbooks and the value of hamster populations and their place of belonging were rethought. Populations were less and less defined in terms of spatially bound bodies, but more and more in terms of freely circulating genes. The space where animals actually lived no longer mattered, only the space where they (or their forebears) originated from.

Captivity, of course, limited hamster agency in various ways. Human planning, for instance, replaced natural migrations and partner choice. Yet, even in the captive program the physical and behavioural attributes of the hamster continued to matter. Food preferences, menstrual cycles and hibernation periods shaped the ways in which breeding was organized. At times, furthermore, unexpected hamster behaviour obliged a ‘renegotiation’ of the program. Breeders, for instance, had to adapt their practices in response to the aggressive reactions of females vis-à-vis males – of which two had gotten killed in Rotterdam zoo (“Twee Rotterdamse korenwolven overleden” 2000). Only by taking hamster behaviour seriously, a successful biopolitics of breeding could gradually take shape. And successful it was. Within just a few years there were more than enough hamsters to start reintroducing them into the wild.

## Choreographies of Reintroduction

As indicated, the ex situ breeding of European hamsters was not evident. Yet, once translated into genetic terms, the factors to be controlled also proved relatively limited. Controlling a reintroduction, however, was more complex of an issue. It involved getting a grip on the hamster’s interaction with entire landscapes and the range of human and non-human actors that resided there. In order to make the biopolitics of reintroduction into a success, its supervisors needed to come up with – what soon turned out to be – highly complicated choreographies.

Reintroduction quickly proved a bone of contention between the partners of the task force. Early plans contained ambitious goals of setting up core hamster reserves to be connected through corridors and supplemented with areas under ‘adjusted’ agricultural regimes (Krekels 1999; Krekels et al. 1996). Yet, since hamsters prefer fertile plots that contain high yields, land for reserves proved both hard to find and expensive. With the Secretary of State only slowly acquiring hamster habitat in 2001 and 2002, *Das en Boom* complained that, while the numbers in their breeding centre increased, no viable space for reintroduction was available. In the press, Dirkmaat indicated that the Secretary of State was pushing the reintroduction for ‘quick electoral success’, but that *Das en Boom* would refuse to take part as long as there was no suitable habitat available (“Gefokte korenwolfjes” 2002). He added that, if no habitat was found, he would release his hamsters at the Inner Court of the Ministry of General Affairs in The Hague, or turn them into a fur coat for the Princess of Orange (Coenradie 2002). Despite bickering in the media, a compromise was eventually agreed upon. In April 2002, two hamsters were brought in from Rotterdam to Limburg to be photographed by the press. The Secretary of State, also aware of the power of anthropomorphizing, presented them as ‘Floris’ and ‘Fatima’ – in this way providing the name-giving with a multi-cultural touch. Despite the will of national authorities to move quickly, a provincial civil servant convinced them to postpone the actual reintroduction until summer, when wheat would provide the hamsters with some cover (Schreuder 2002). After a habituation period in a release pen, 44 individuals were eventually released into the fields in July, where they were provided with ‘artificial burrows’. Over the following years, more reintroductions followed at an increasing number of locations (Haye 2006; Haye et al. 2005).

Despite the compromise over the reintroduction, the relations between *Das en Boom* and the Secretary of State remained strenuous and in 2005 the organization retreated from the program. In the newspapers, Dirkmaat indicated that, with only three out of eleven promised reserves established, the saving operation had turned into ‘end-of-life care’ (“Korenwolf doet het niet” 2005). Looking back on the episode today, he adds that, by 2005, *Das en Boom* had actually lost control over the situation. The government had decided to move the hamster breeding from their facility in Beek-Ubbergen to the newly established Limburg zoo Gaia Park with the expressed goal to create a local ‘support base’. With the management over the breeding, *Das en Boom* had, in Dirkmaat’s view, lost its most important means to bring pressure to bear (J. Dirkmaat, personal communication, March 9, 2020).

Dirkmaat’s stark pessimism stood in contrast to the cautious optimism of the biologists engaged in the project, who, at least in the initial years, saw the number of hamsters in the Limburg fields increase (Haye 2006). Their understanding of the reintroduction success was partially based on the traditional method of counting burrows, but they also developed new techniques. Following the guidelines of the International Union for the Conservation of Nature, the scientific leaders of the project invested significantly in post-release monitoring (Krekels 1999). Like in the case of other secretive animals (Benson 2010), they opted for telemetry to render the nocturnal and subterraneous hamster visible. Each year, veterinarians inserted radio-trackers in the abdominal cavity of 30 to 60 reintroduced hamsters, allowing to analyse their movements and survival (Haye et al. 2005, p. 8). Later, the tinkering of local technicians even led to the development of a tracker that allowed for the detailed measurement of body temperatures. This enabled the project members to monitor from a distance when female hamsters were fertilized, when young were born and when lactation stopped, but also when individuals were hibernating or when they were killed (G. Müskens, personal communication, February 6, 2020). Slowly and like other animal populations in high-profile conservation projects, the Limburg hamster became subject to a ‘panoptic surveillance assemblage’ (Lorimer 2015, p. 82).

While hamster numbers went up, the results of hamster monitoring also showed some worrying trends. Reintroduced individuals and particularly males, turned out to have low survival rates. According to the researchers, the released hamsters needed three to four weeks to adapt to their new environment – a period in which they proved particularly vulnerable to predation (Haye et al. 2005). Furthermore, they argued that hamster reserves, surrounded by fields with intensive agriculture, constituted ‘a kind of oasis in a relatively empty desert and as such [were] an important magnet for predators’ (Haye et al. 2005, p. 31). Analyses showed that particularly foxes found their way to the reintroduced individuals, claiming up to 44% of the deaths of male hamsters and 33% of the female ones (Haye et al. 2008, p. 188). Some foxes were even known to purposely hang out in the vicinity of hamster release cages (Pillot 2003, p. 32). The press got word of this and was quick to conclude that the ‘hamster paradise’ was nothing but ‘a snack bar for foxes’(Schreuder 2004). The journalists, furthermore, did not shy away from calculating how much tax money the foxes’ snacks were actually worth (“Vos trekt zich niets aan” 2005).

Partially because of negative coverage in the press, the high predation rate was considered problematic and the Province of Limburg decided to hire a fox expert for consultation. In a first stage, the consultant experimented with offering dead chickens as ‘distraction feeding’ to lure the foxes away from hamster reserves (Mulder et al. 2003, p. 27). This proved unsuccessful. In a second stage, a two-year research project was launched in which foxes were wired and the effects of targeted hunting were monitored. On the basis of the experiment, then, the consultant could conclude that hunting was inefficient for most of the year as foxes quickly tended to occupy vacant territories. The most viable approach, he indicated, was to shoot adult foxes in winter, when they were least mobile, so that their territories would remain empty until hamsters were released in spring. Furthermore, as an accompanying measure, he suggested to put up electric fences around the reintroduction sites (Mulder 2007). Over the following years, both suggestions were implemented in an attempt to keep hamsters and foxes at least partially spatially separate (Haye et al. 2008). The press, however, remained sceptical. One journalist indicated the whole project showed a typically Dutch ‘strive for makeability’ in a context of ‘minimal manoeuvring space’(Somers 2010).

Fine-tuning the choreography of reintroduction not only consisted of spatially organizing hamsters and their predators, but also of managing human activities. From the first plans in 1999, the goal of the hamster experts was to combine reserves with wider areas in which farmers would be subsidized to institute ‘hamster-friendly agriculture’. Yet, this approach proved difficult on various accounts. Farmers were reluctant to sell their best lands for reserves and, many of them proved suspicious of the zoologist-designed schemes for hamster-friendly agriculture (G. Müskens, personal communication, February 6, 2020). The fact that agricultural organizations spurred this suspicion did not help, nor that local farmers were unrepresented in the Hamster Consultation Group Limburg [*Hamster Overleg Limburg*], which apart from governmental officials consisted of scientists and representatives of conservation societies. As a result, hamster policies gained a reputation for their ‘top-down’ and ‘unpractical’ character (B. van Noorden, personal communication, March 12, 2020). Aware of this problem, the province carried out an institutional reorganization when it took over the hamster dossier from the national government in 2005. It turned the advisory Consultation Group into an executive Hamster Commission [*Korenwolfcommissie*] and included local farmers and hunters, alongside biologists and conservationists. Increased subsidies and more flexible management arrangements were agreed upon and helped to attract new participating farmers (“Aantrekkelijke subsidies” 2006; “Verbeterde beheersafspraken” 2007). Local embedding and identity-building became more important. Since 2007, the yearly information event of the Commission introduced a farmer’s prize for ‘best management’ and, in 2011, a prize was added for the farmer with the most burrows on his land. The latter prize was sponsored by a local brewery that, since the mid-1990s, marketed its own ‘hamster beer’ (“De Gulpener Bierbrouwerij” 2013).

Creating an effective ‘hamster-friendly’ agriculture, however, went beyond the social dimension of engaging farmers. It also involved coming up with a management regime that re-aligned agricultural practice with hamster life cycles, providing them with year round cover and food. Zoologists deemed the continuing expansion of maize cultivation as detrimental and they were convinced that increasingly early and efficient cereal harvesting interfered with the hamster’s second litter. The Hamster Commission therefore pushed subsidies for a shifting cultivation of winter grain, summer grain and alfalfa, for postponing harvest until September and for leaving some strips or, in some cases, entire fields unharvested (Haye et al. 2005; Haye Koelewijn et al. 2014; Haye Swinnen et al. 2014; Kuiters et al. 2010). The details of the management were subject to continuous tinkering however. An early ban on herbicides and fertilizers, for instance, seemed to backfire as crops apparently became too sparse to give cover and crucial hamster food was overgrown with weeds (Kuiters et al. 2010). It turned out that areas deemed ‘natural’ (with foxes and without herbicides) were not necessarily the spaces in which hamsters thrived.

Despite the measures taken, hamster populations failed to become self-sustaining. After a highpoint of an estimated 500 individuals in 2007, populations steeply dropped in 2008 and stayed low since. In an attempt to stem the tide, the Commission responded with new strategies. In small territories, the hamsters proved vulnerable of becoming locked in. Hamster reserves were therefore reconceptualised as ‘stepping stones’ in larger landscapes, of which a planned total of 25% should come under hamster-friendly management. The project ‘Hamster on its own feet’ [*Hamster op eigen benen*], launched in 2015, ambitiously aimed for three clusters of at least 250 hectares. In order for the hamster to survive in such a dynamic landscape, in which crops might switch yearly, the project’s taskforce also recommended dividing up the plots that had become increasingly extensive because of agricultural market concentration. Plots of less than five hectares, so the policy plan of 2015 reads, would be small enough to enable hamsters to ‘move house’ when crops changed (Müskens et al. 2019, p. 68). Such landscape interventions, the Commission indicated, not just benefited one species. With hamster populations at continuously low levels, outward communication made sure to stress the overall biodiversity benefits of the measures taken, including the effects for (widely popular) farmland birds, cornflowers and poppies (Cerfontaine et al. 2020; Kuiters et al. 2010, p. 49; Müskens et al. 2018, p. 20). In the Commission’s representation the European hamster became a ‘guiding species’ as well as a symbol of ‘agrarian nature management’(Kossen 2020).

The ‘Hamster on its own feet’ project not only lobbied to redesign the agricultural landscape, but also to adapt the mechanical means of cultivating it. More in particular, it launched experiments with stripper combines that harvested the ears of the grain, but left the straw standing. This rather old-fashioned technology was relatively widespread in the US and Australia, where large scale production and low value of straw, rendered it a more efficient than alternative harvesting machines. The benefit for hamster management would be that it provided the farmer with a harvest, while offering a continued cover for the hamster against predators. As such, it would enable an extension of hamster-friendly agriculture over a larger territory for the same subsidy cost (Müskens et al. 2019). Ecologists further liked that the strippers were ‘messy’, spilling quite some grain that could serve the hamsters as food (G. Müskens, personal communication, February 6, 2020). The stripper, thus, was hailed for its potential of changing the choreography between humans and non-humans once again.

It might be clear that the high-profile reintroduction of hamsters in Limburg was a project of widening proportions. Whereas the captive breeding of the European hamster came with relatively straightforward biopolitics, a successful reintroduction of the species entailed the mastering of choreographies involving ever more actors. Consultants, commissions and taskforces were engaged in what amounted to a multispecies negotiation process. To be sure, farmers and politicians needed to be enrolled as participants in the program, but so were non-human actors such as foxes and hamsters. In order to re-establish the latter in the Limburg landscape, a continuous tinkering was required, taking into account hamster preferences for fertile plots, the adaptation period they needed after release, their life cycles and their needs in terms of food and cover. The secretive and underground lifestyle of hamsters, furthermore, made that their ‘demands’ could only be heard at great effort, if at all. For all these reasons, the success of hamster reintroduction remained relative and vulnerable. In their personal communications, several participants in the reintroduction program referred to the limits of their power in a wider context of intensifying agriculture, growing political impatience and declining hamster fertility (G. Müskens, personal communication, February 6, 2020; M. la Haye, personal communication, March 3, 2020; B. van Noorden, personal communication, March 12, 2020). Clearly, the biopolitical control they exerted was fragile. Until today, results remain uncertain.

## Epilogue

On 10 October 2018, Gaia Zoo (the former Gaia Park) opened the doors of its Limburg House. Presented as an ‘Ark of Noah’, it showcased threatened animals typical of the province. Apart from the fire salamander and the garden dormouse, the European hamster received a prominent place. During the festive opening, at which ‘hamster beer’ was served, the zoo director made references to local identity-building and responsibilities of environmental stewardship. The Zoo, so it was claimed, had a moral obligation for the survival of the animals of the province (Haye et al. 2018). The Limburg House, of course, only constitutes a tiny part of the biopolitical assemblage that has to keep the hamster from extinction. This assemblage further consists of international agreements (and the potential fines these can bring), breeding centres, genetic theories, farming subsidies, artificial burrows, anti-fox fences and stripper combines. Taken together, they at least partially determine the rhythm of the multispecies choreographies in the fields of Dutch Limburg.

As this article has shown, a century and a half of Dutch biopolitical engagement with the European hamster generated diverse often contradictory discourses and practices. From an invasive and aggressive pest cast in military metaphor, the hamster population in Dutch Limburg was turned into (and managed as) a useful symbol of the traditional landscape, a genetically poor lineage and a guiding species of sustainable farming. These representations are partially reflective of the changing expert cultures of agricultural scientists, zoologists, geneticists and ecologists, but they were equally shaped by the actions of journalists, activists and farmers. The evolving discourses fed into practices of killing, breeding and fostering, which, in turn, were enabled by instruments such as maps, studbooks and radio-trackers. Particular forms of biopolitics were, furthermore, co-constructed with specific spaces: a modern landscape of production (where the hamster was unwelcome), a disappearing traditional countryside (of which it constituted the marker), a deterritorialized space of breeding (in which it could genetically ‘recover’) and a land of cohabitation (in which its doings were to be harmonized with those of foxes and farmers).

While notions about the indigeneity of the European hamster changed over time, it was uninterruptedly cast and managed as a ‘wild’ species. Its distant relative *Mesocricetus aureus,* to the contrary, was conceived, bred and circulated as a ‘domesticated’ animal. Historically, both species moved through different geographies, ecologies and relations to humans. While the first travelled from the Eurasian steppes via the modernizing agricultural landscapes of nineteenth-century Europe to end up in breeding centers and hamster-friendly fields, the second moved from the semi-desert in the French mandate of Syria and Lebanon to British interwar laboratories and, then, to Dutch pet cages. Such trajectories clearly resist the dichotomy between wild and domesticated. Through their histories, both *Mesocricetus aureus* and *Cricetus cricetus* show themselves as ‘hybrids’ inhabiting ever changing nature-cultures. As far as the latter is concerned, the trajectory has certainly not come to an end either. In Eastern and Central Europe, European hamsters are increasingly spotted in urban gardens and parks – a process biologists describe as synurbanization (Feoktistova et al. 2016; Surov et al. 2016). Ironically, these urban hamsters largely seem to escape the intricate regimes of biopolitical control as they have been developed in the Netherlands.

This brings us to the question how we can understand the agency of European hamsters in the context of changing biopolitical regimes. It is not hard to see, I believe, that hamsters ‘made a difference’ – either by destabilizing, transgressing or resisting those regimes, or by influencing, enabling or sustaining them. In the late nineteenth century, hamsters transgressed the orderings of productive agriculture and, at least for a while, escaped the control of agronomists. Around 2000, their physical and behavioural attributes proved crucial for enabling (and ‘renegotiating’) breeding programs to ‘save’ their population. In the twenty-first century, then, they regularly defied biopolitical expectations – dodging herbicide-free fields or clumsily running into foxes after release. Throughout, their secretive behaviour substantially complicated human observation and, thus, scientific understanding and control. Of course, as Mieke Roscher reminds us, animal agency should always be understood as fundamentally relational (Roscher 2019). In the case of the European hamster, like that of other animals, it was continuously influenced by changing human technologies, representations and policy regimes.

What this paper ultimately sought to stress is that hamster agency at least partially resided in its numbers. Quantities mattered for the ways in which hamsters effected change, whether they were considered too numerous or too rare. Their population explosions in specific late-nineteenth-century landscapes (alongside those of other ‘pests’) were instrumental in the development of agricultural science. Their implosion in the late twentieth century (alongside that of other ‘wild’ creatures) created a sense of urgency for local activists to push for new biopolitics of managing wildlife. The relative ease with which numbers picked up once in breeding centres enabled particular regimes of ex situ conservation. The dwindling numbers after reintroduction, finally, raised question marks over the long-term prospects of cohabitation with humans and forced the Hamster Commission to continuously tinker with their plans.

The paradox, of course, is that the less European hamsters remained, the more humans they ultimately mobilized. Hamsters made a difference because of their quantities, but also because of the human values attached to those quantities. Even power in numbers, so it seems, is ultimately relational.
